# Risk Factors for Hospital Readmission Following Noncardiac Surgery: International Cohort Study

**DOI:** 10.1097/AS9.0000000000000417

**Published:** 2024-04-09

**Authors:** Michael H. McGillion, Flavia K. Borges, David Conen, Daniel I. Sessler, Brenda L. Coleman, Maura Marcucci, Carley Ouellette, Marissa Bird, Carly Whitmore, Shaunattonie Henry, Sandra Ofori, Shirley M. Pettit, Deborah M. Bedini, Leslie P. Gauthier, Jennifer Lounsbury, Nancy M. Carter, Vikas Tandon, Ameen Patel, Teresa Cafaro, Marko R. Simunovic, John A. Harlock, Diane Heels-Ansdell, Fadi Elias, Theodore Rapanos, Shawn Forbes, Elizabeth Peter, Judy Watt-Watson, Kelly Metcalfe, Sandra L. Carroll, Philip J. Devereaux

**Affiliations:** * From the School of Nursing, McMaster University, Hamilton, ON, Canada; † Population Health Research Institute, Hamilton, ON, Canada; ‡ School of Medicine, McMaster University, Hamilton, ON, Canada; § Health Research Methods, Evidence, and Impact, McMaster University, Hamilton, ON, Canada; ‖ Outcomes Research Consortium, Cleveland Clinic, Cleveland, OH; ¶ Sinai Health System, Toronto, ON, Canada; # Dalla Lana School of Public Health, University of Toronto, Toronto, ON, Canada; ** Hamilton Health Sciences, Hamilton General Hospital, Hamilton, ON, Canada; †† Hamilton Health Sciences, Juravinski Hospital, Hamilton, ON, Canada; ‡‡ Department of Surgery, McMaster University, Hamilton, ON, Canada; §§ Bloomberg Faculty of Nursing, University of Toronto, Toronto, ON, Canada.

**Keywords:** cohort study, hospital readmission, noncardiac surgery, risk factors

## Abstract

**Objective:**

To determine timing and risk factors associated with readmission within 30 days of discharge following noncardiac surgery.

**Background:**

Hospital readmission after noncardiac surgery is costly. Data on the drivers of readmission have largely been derived from single-center studies focused on a single surgical procedure with uncertainty regarding generalizability.

**Methods:**

We undertook an international (28 centers, 14 countries) prospective cohort study of a representative sample of adults ≥45 years of age who underwent noncardiac surgery. Risk factors for readmission were assessed using Cox regression (ClinicalTrials.gov, NCT00512109).

**Results:**

Of 36,657 eligible participants, 2744 (7.5%; 95% confidence interval [CI], 7.2–7.8) were readmitted within 30 days of discharge. Rates of readmission were highest in the first 7 days after discharge and declined over the follow-up period. Multivariable analyses demonstrated that 9 baseline characteristics (eg, cancer treatment in past 6 months; adjusted hazard ratio [HR], 1.44; 95% CI, 1.30–1.59), 5 baseline laboratory and physical measures (eg, estimated glomerular filtration rate or on dialysis; HR, 1.47; 95% CI, 1.24–1.75), 7 surgery types (eg, general surgery; HR, 1.86; 95% CI, 1.61–2.16), 5 index hospitalization events (eg, stroke; HR, 2.21; 95% CI, 1.24–3.94), and 3 other factors (eg, discharge to nursing home; HR, 1.61; 95% CI, 1.33–1.95) were associated with readmission.

**Conclusions:**

Readmission following noncardiac surgery is common (1 in 13 patients). We identified perioperative risk factors associated with 30-day readmission that can help frontline clinicians identify which patients are at the highest risk of readmission and target them for preventive measures.

## Abstract 2

Mini abstract:

We undertook an international prospective cohort study of adults ≥45 years of age who underwent noncardiac surgery to determine risk factors associated with readmission. A number of risk factors were identified that can help frontline clinicians target patients at the highest risk of readmission for preventive measures.

## INTRODUCTION

Globally, over 100 million major noncardiac surgeries are performed annually on adults over the age of 45.^1,2^ While surgery is critical to optimizing global health and well-being, it is associated with complications and unplanned hospital readmissions.^3^ Studies of 30-day readmission have reported rates ranging from 5.7% to 18.5%.^4–9^

Drivers of readmission are multifactorial. At the patient level, a number of baseline factors have been identified, including age, sex, body mass index (BMI), and baseline conditions such as pulmonary, heart, renal, or liver disease, diabetes, or active or recent treatment for cancer.^6,10,11^ Risk of readmission following noncardiac surgery has also been associated with a number of in-hospital adverse events including vascular, infectious, and hemodynamic complications, surgical reintervention, prolonged length of stay, and intensive care unit admission.^6,8,12,13^ Limitations of these data are that they are older and have been derived from single-center studies focused on a single surgical procedure with uncertain generalizability. To enhance our understanding of risk factors for readmission, large cohort studies are needed that include multiple surgical procedures across several surgical centers internationally.

In this substudy of the international Vascular Events in Noncardiac Surgery Patients Cohort Evaluation (VISION) study,^14–16^ we systematically followed patients who underwent noncardiac surgery and documented perioperative complications and 30-day readmission after discharge. Our primary objectives were to determine the rate and risk factors associated with the 30-day readmission of these patients. A secondary objective was to develop a risk-based score to predict readmission of adult patients undergoing noncardiac surgery, with the aim of facilitating decision-making about who may need preventive follow-up.

## METHODS

### Study Design and Eligibility Criteria

VISION was a prospective cohort study^14–17^ that enrolled a representative sample of adult patients from 28 academic hospitals in 14 countries from 2007 to 2013 (ClinicalTrials.gov, NCT00512109). The study received approval from the research ethics boards at each site before enrollment started. All patients or their substitute decision-makers gave written informed consent either before surgery or within 24 hours for emergent, urgent, or night surgeries. A deferred consent was used in 9 centers for patients unable to provide consent and for whom no substitute decision-maker was available.^17^

Details of the study design and methods are available elsewhere.^14,17^ In short, adult patients (≥45 years old) were eligible if they underwent inpatient noncardiac surgery, received either general or regional anesthesia, and stayed at least one night in hospital after surgery. Patients were not eligible for enrollment if they refused consent or were previously enrolled in VISION.^14,17^ For these analyses, participants were excluded if they died during their index hospitalization, were not discharged before day 30 after surgery, or were lost to follow-up.

Consenting patients were interviewed and charts were reviewed to collect baseline and in-hospital data. Patients were contacted after surgery to inform 30-day outcomes, including hospital readmission.

The primary outcome of this study was hospital readmission within 30 days of hospital discharge following the index surgery. If more than one readmission occurred within 30 days, the one due to a vascular reason (ie, myocardial infarction, cardiac arrest, stroke, congestive heart failure, ischemic symptoms with ST or T wave changes on an ECG, cardiac arrhythmia, cardiac revascularization procedure, deep venous thrombosis, pulmonary embolus, any vascular surgery, or bleeding) was recorded on the case report form. If more than one readmission was due to a vascular reason, the longer/longest length of stay was recorded.

Covariates considered as potential risk factors for readmission are listed in Supplemental Table 1, see http://links.lww.com/AOSO/A316. Detailed subtypes of surgery are listed in Supplemental Table 2, see http://links.lww.com/AOSO/A316. Anemia was defined as a preoperative hemoglobin value <120 g/L for women and <140 g/L for men.^18^ Values for the following measures were recorded only if they fell within prespecified abnormal ranges including hypoxemia: blood oxygen saturation (SpO_2_) <90%, low: <100 mm Hg or high: >160 mm Hg systolic blood pressure, and bradycardia: <55 or tachycardia: >100 beats/min. Hypoxemia, measured through pulse oximetry, was recorded only after extubation in patients who were intubated for surgery. The duration of hypoxemia was defined as the total time of all hypoxemic events with the cut point determined using the value most predictive of readmission (ie, 20 minutes). Myocardial injury after noncardiac surgery (MINS) was measured from surgery to 30 days following surgery.^14^ Height and weight were converted to BMI categories. Estimated glomerular filtration rates (eGFR) were calculated using the abbreviated Modification of Diet and Renal Disease formula (excluding body surface area).^19^

### Statistical Analyses

The percentage of patients readmitted within 30 days of discharge was calculated using the number of patients discharged alive with complete 30-day follow-up and their first readmission to an acute care hospital. Missing values for baseline and baseline physical measures were imputed using linear or logistic regression and age, sex, and center as predictive variables.^20^ A sensitivity analysis was conducted to assess the impact of imputation.

Cox proportion hazard regression with robust variance estimation was used to estimate hazard ratios (HRs) for the first hospital readmission adjusting for contributing factors and confounding variables.^21^ Models were constructed using all contributing factors that were removed, one at a time, starting with those with the largest *P* value until all variables were associated at a value of ≤0.05. All variables were added back, one at a time, to the reduced model. They were retained if they were associated at a *P* value of ≤0.05.^22^ Biologically plausible effect measure modifiers were then assessed (eg, baseline and postoperative blood pressure, heart rate). The model was tested for compliance with the proportional hazards assumption.

To develop the risk-based scores, HRs were used. Risk scores were calculated using the methods set out by Sullivan et al.^23^ The risk for readmission associated with a 12.5-year increase in age was set to one point in the overall risk score.^23^ Higher sums of individual points predict a higher risk of readmission.

Population attributable fractions (PAF), adjusted for other variables in the model, were calculated using the *punafcc* command following Cox regression analysis of covariates selected in the main model.^24^ All analyses were conducted using Stata SE version 14.2 (College Station, Texas).

## RESULTS

**FIGURE 1.**
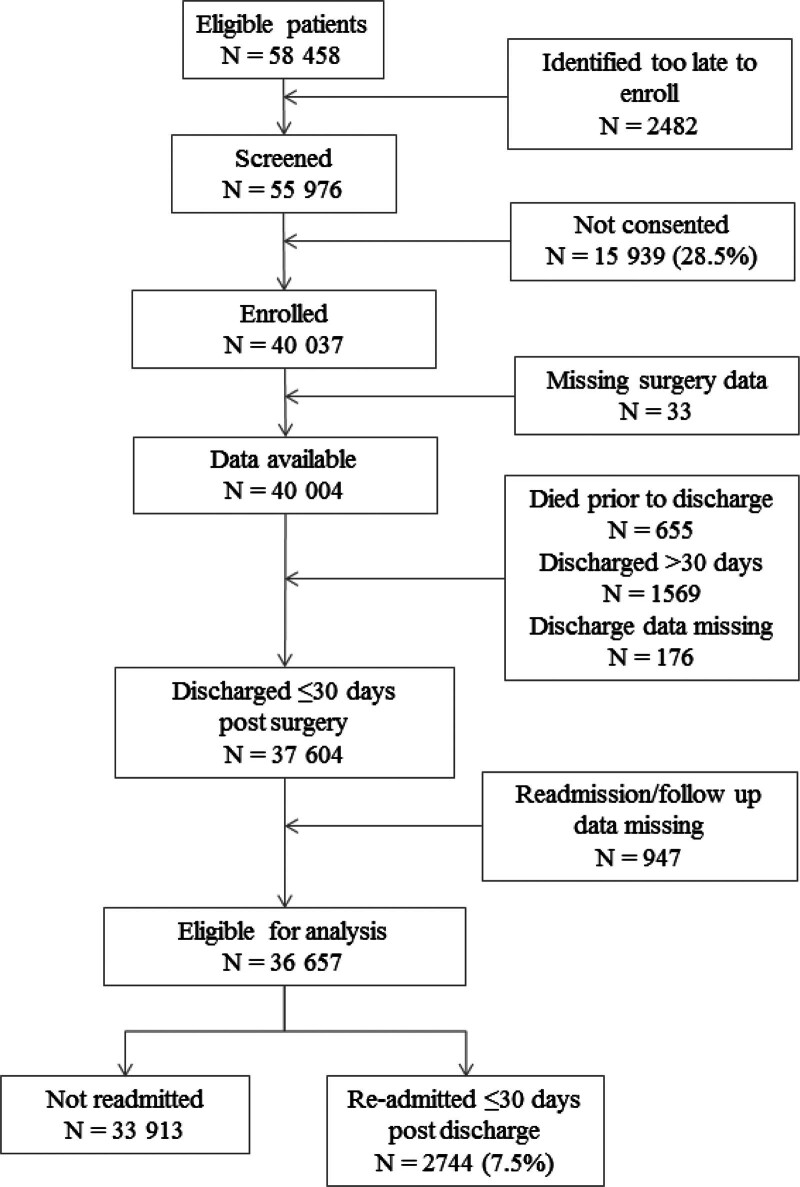
Flow chart of participants in VISION 30-day readmission following discharge.

As shown in Figure 1, 36,657 patients were included in these analyses. Participants ranged in age from 45 to 103 years (median, 63.1 years; interquartile range [IQR], 54.8–72.3) and a median length of index hospital stay of 4 days (IQR, 2–7). As shown in Supplemental Table 1, see http://links.lww.com/AOSO/A316, 18,376 (50.1%) participants were female, 3.4% were underweight while 3.7% had a BMI ≥40, 11,053 (30.1%) were enrolled from North American hospitals, and 36.2% underwent low-risk surgeries. While 4274 (11.7%) patients in the study experienced MINS while in hospital, some other postsurgical vascular complications were rare ranging from 0.1% for stroke to 0.4% for deep vein thrombosis/pulmonary embolism. Other major postsurgical complications observed in hospital included pneumonia (0.6%), sepsis (2.2%), and major bleeding (12.8%).

The overall crude rate of first readmission within 30 days of discharge was 7.5% (2744 of 36,657; 95% confidence interval [CI], 7.2–7.8). Supplemental Table 1, see http://links.lww.com/AOSO/A316 presents the crude rates of readmission that range from 5.3% following orthopedic surgery to 9.8% following general surgery and from 6.8% in participants aged 45 to 69 years to 9.1% in patients aged ≥70 years. They also vary across study regions, from 9.7% in Asia to 5.7% in South America (*P* < 0.001). Patients who were readmitted had a longer median length of index hospital stay (6 days; IQR, 3–9) compared with those who were not readmitted (4 days; IQR, 2–7; *P* < 0.001).

**FIGURE 2.**
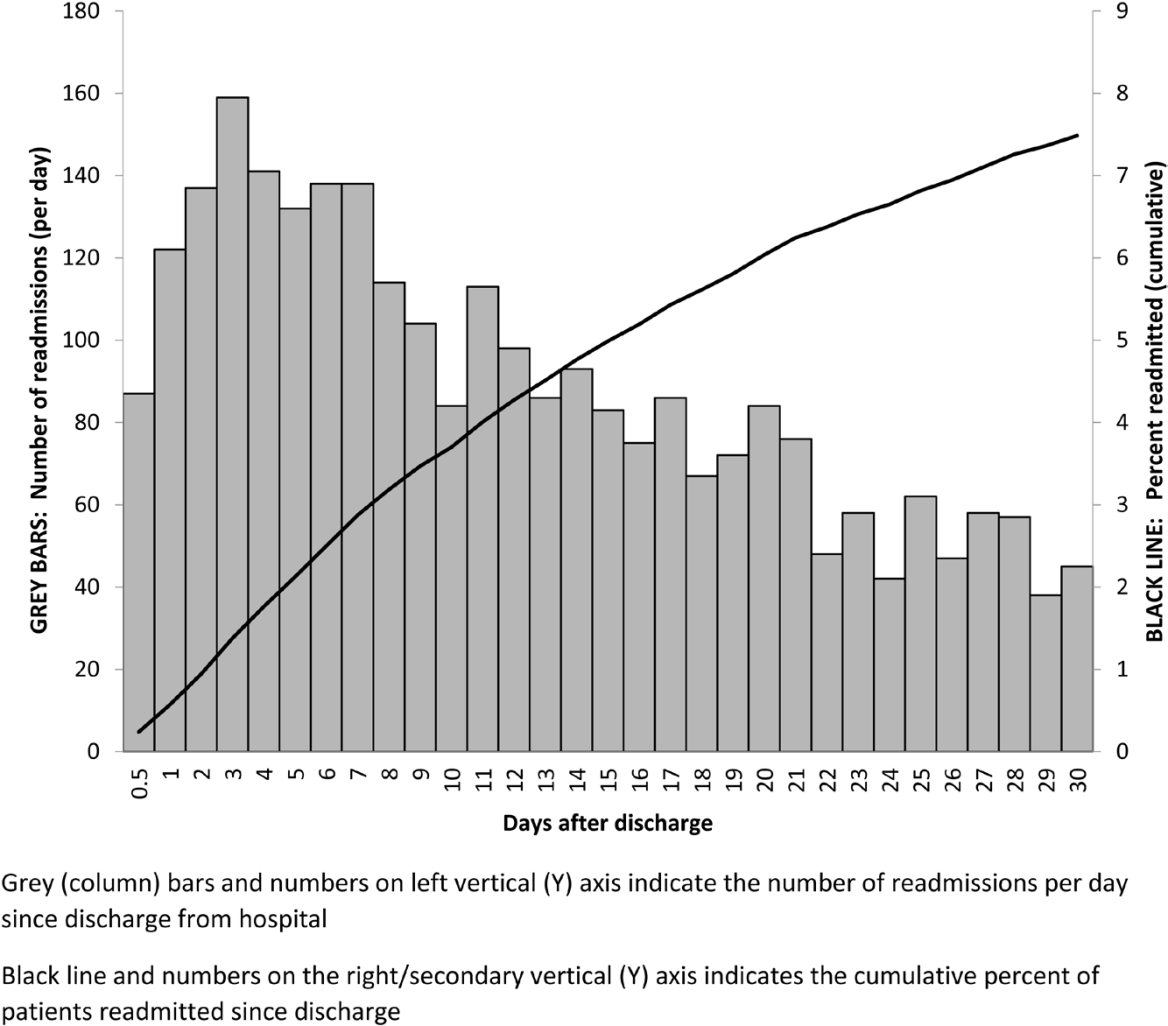
Number and cumulative percent of patients readmitted to hospital within 30 days of discharge following noncardiac surgery. Gray (column) bars and numbers on left vertical (*y*) axis indicate the number of readmissions per day since discharge from hospital. Black line and numbers on the right/secondary vertical (*y*) axis indicate the cumulative percent of patients readmitted since discharge.

Figure 2 shows the number of cases and cumulative rate of readmission to 30 days postdischarge. Rates of readmission were highest in the first 7 days after discharge and declined progressively over the follow-up period.

### Risk Factors for Readmission

Supplemental Table 2, see http://links.lww.com/AOSO/A316 reports baseline demographic and medical history factors associated with readmission within 30 days of discharge in the multivariable model. They included age ≥70 years (adjusted HR, 1.18; 95% CI, 1.09–1.29), having cancer that was treated within the previous 6 months (1.44; 95% CI, 1.30–1.59), a history of tobacco use (1.22; 95% CI, 1.13–1.33), venous thromboembolism (1.28; 95% CI, 1.07–1.59), congestive heart failure (1.23; 95% CI, 1.03–1.46), needing assistance with activities of daily living (1.22; 95% CI, 1.04–1.42), peripheral vascular disease (1.18; 95% CI, 1.01–1.39), coronary artery disease (1.17; 95% CI, 1.05–1.31), and stroke/transient ischemic attack (1.15; 95% CI, 1.00–1.32).

Baseline laboratory and physical measures associated with readmission included anemia (HR, 1.28; 95% CI, 1.18–1.39), eGFR <30 mL/min/1.732 or receiving dialysis (1.47; 95% CI, 1.24–1.75), low systolic blood pressure (1.38; 95% CI, 1.09–1.74), and a BMI of <18.5 (1.11; 95% CI, 1.02–1.21). Patients with tachycardia (1.57; 95% CI, 1.19–2.08) or a normal heart rate (1.35; 95% CI, 1.06–1.71) were at higher risk of readmission than patients with bradycardia (Supplemental Table 2, see http://links.lww.com/AOSO/A316).

After adjusting for baseline demographic and laboratory and physical measures, events that occurred during the index hospitalization that remained significantly associated with the risk of readmission included type of surgery, with all types of surgery having a higher risk of readmission than orthopedic surgeries (Supplemental Table 2, see http://links.lww.com/AOSO/A316). Other variables included stroke (HR, 2.21; 95% CI, 1.24–3.94); hypoxemia (1.36; 95% CI, 1.12–1.65); heart rates >100 beats/min (1.22; 95% CI, 1.10–1.35), below 55/min (1.15; 95% CI, 1.03–1.27), or episodes of both tachycardia and bradycardia (1.42; 95% CI, 1.23–1.64); and a major bleed (1.19; 95% CI, 1.07–1.33) either during or after surgery. Patients who had MINS within 30 days of surgery (HR, 1.17; 95% CI, 1.05–1.31), a postsurgery length of stay of ≥4 days (1.38; 95% CI, 1.26–1.50), and patients discharged to a nursing home/long-term care facility (1.61; 95% CI, 1.33–1.95) were also at higher risk of admission. The C-statistic/area under the ROC curve for the model was 67.2% (*P* < 0.01). A sensitivity analysis revealed no clinically significant differences in risk factor results when restricting the analysis to the 27,963 observations without imputed values.

Sixty patients (0.16%) died after discharge but during the 30-day follow-up period and after readmission to hospital. The mortality rate was higher for those readmitted ≤3 days (3.6%) compared with ≥4 days (1.5%) after discharge (*P* = 0.002).

Supplemental Table 2, see http://links.lww.com/AOSO/A316 also shows the adjusted PAF for factors associated with readmission. Several of the adjusted PAFs were modest. The highest PAFs included length of index hospitalization ≥4 days after surgery (13.3%; 95% CI, 9.6–16.4), low-risk surgery (10.1%; 95% CI, 7.3–12.7), baseline anemia (9.7%; 95% CI, 6.7–12.8), general surgery (9.4%; 95% CI, 7.3–11.1), and a history of tobacco use (8.0%; 95% CI, 4.9–11.0). When stratified by surgery type (Supplemental Table 3, see http://links.lww.com/AOSO/A316), the PAFs of patients undergoing general surgeries were significantly higher for those with lengths of stay longer than 4 days (29.4; 95% CI, 18.4–39.0) compared with patients undergoing other types of surgery. Stratification by study region (Supplemental Table 4, see http://links.lww.com/AOSO/A316) highlighted some significant differences in PAF by covariate for readmission for patients across continents (eg, baseline tobacco use). Stratification by year of surgery (Supplemental Table 5, see http://links.lww.com/AOSO/A316) showed no significant differences over the 3 intervals examined (ie, 2007–2009; 2010–2011; 2012–2013).

### Model-Based Risk Score for Readmission

The model-based risk score for readmission yielded points for each covariate ranging from 1 for underweight patients to 12 for patients who had a stroke while in hospital (Supplemental Table 2, see http://links.lww.com/AOSO/A316). For example, although only 44 patients (0.1%) had a stroke while in hospital, 27.3% of those who did were readmitted within 30 days of discharge compared with 12.7% of patients who were underweight. As shown in Supplemental Table 6, see http://links.lww.com/AOSO/A316, the estimated risk of readmission based on the formulae suggested by Sullivan et al^23^ was 5.1% for patients whose factors summed to 15 points.

## DISCUSSION

In our international, multicenter study of 36,657 patients who underwent inpatient noncardiac surgery, the rate of readmission to hospital within 30 days of discharge was 7.5% but was significantly higher for frail older patients with a history of medical issues. In addition, patients who had a stroke, major bleed, MINS, a longer length of stay, in-hospital heart rate abnormalities, or hypoxemia were at higher risk of readmission. We demonstrated that the rates of readmission were higher in the first 7 days after discharge. We also developed a scoring system to identify risk factors for readmission that require external validation.

Two studies of the US National Surgical Quality Improvement Program database reported that 6.1% and 6.8% of patients undergoing noncardiac surgery were readmitted within 30 days of surgery, which is similar to the 7.5% we observed.^4,8^ In our study, rates of readmission varied by type of surgery, from 5.3% to 9.8% following orthopedic and general surgeries, respectively. Similar rates were reported in other studies, with rates of readmission being lower following orthopedic surgery (4.5%–6.3%)^8,10,25,26^ than following general surgeries (5.9%–13.6%).^6,9,25,27–29^ In examination of our data stratified by surgery type, patients undergoing general surgery with a length of stay of 4 or more days had a significantly higher PAF for readmission compared with other types of noncardiac surgery. These data suggest that these patients may be a priority for supportive interventions such as hospital-to-home remote monitoring and virtual care.^30^

Given that over 100 million adults ≥45 years worldwide undergo noncardiac surgery annually,^1,2^ our results suggest that 7.5 million of these adults are readmitted to an acute care hospital. In our study, 38.4% of readmitted patients were readmitted within 7 days. The daily rate of readmission was approximately 0.4% during the first week, 0.3% in the second week, and 0.2% for days 15–30. Our findings are similar to those published by Lefevre et al,^7^ who reported a decline in the rate of readmission following all surgery types in French hospitals, but their rate remained at 0.2% of patients per day from days 8 through 30.

Our risk scores highlight factors most highly associated with readmission in our noncardiac surgery population. The highest score (ie, 12) was for in-hospital stroke. Cantrell et al^31^ reported that the odds of readmission after total hip arthroplasty were 7.6 (2.9–19.9) for patients who suffered a postsurgical stroke compared with those who did not. Of concern, it has been reported that 7% of patients ≥65 years old who underwent noncardiac surgery experienced covert perioperative stroke.^32,33^

Other factors associated with readmission (ie, 4 or 3 points each) in our study included: recent treatment for cancer, low eGFR or being on dialysis, being discharged to a nursing home, baseline tachycardia, low baseline blood pressure, and length of hospital stay ≥4 days. Similar risk factors for readmission have been reported previously including patient characteristics (eg, older age, tobacco use, being underweight, and needing assistance with activities of daily living), underlying health conditions (eg, cancer, cardiovascular disease, anemia, and liver disease), and inpatient events (eg, longer lengths of stay and bleeding/blood loss).^7,8,17,28,29,34^

We also found that patients were more likely to be readmitted if they had postoperative hypoxemia or had postoperative episodes of both tachycardia and bradycardia. We found no other studies that considered these biophysical measures as potential risk factors for readmission. Hypoxemia has been associated with longer lengths of stay in intensive care units postoperatively, a higher risk of impaired wound healing, postoperative myocardial injury, and death.^35–37^ Hypoxemia, tachycardia, and bradycardia are often missed with routine vital signs surveillance. In one study, 90% of hypoxemic episodes (SpO2 <90%) lasting ≥1 hour detected by continuous pulse oximetry were missed by routine vital signs measurements.^37^ Given this scenario, a potential area for future exploration is the use of new automated monitoring technologies. These technologies can monitor patients’ vital metrics continuously and alert clinicians on the ward to impending hypoxemia to facilitate prompt, corrective interventions.^30,38^

Our study has several strengths. The VISION cohort^19^ is a large, international study of a representative sample of adult patients who underwent noncardiac surgery inclusive of multiple surgical procedures across many surgical centers. To our knowledge, this is the largest globally diverse prospective study to examine risk factors for readmission following noncardiac surgery. Our risk factors for readmission were stable over time, and largely similar across countries and regions, providing a rich source of data from multiple centers that is generalizable, as compared to data from single-center studies, or administrative databases, with more limited generalizability.^6,8,12,13^

There are some limitations. Of the 58,458 eligible patients, 40,037 (68.5%) were eligible and consented to participate, raising the issue of participation bias. However, of the 40,037 patients enrolled, 91.6% were included in this analysis. We included only one readmission per patient. It is very likely that our data underestimates the incidence of hypoxemia, tachycardia, and abnormal blood pressure during index hospitalization; whether the episodes recorded were more severe in nature or longer in duration than undetected and/or unrecorded episodes is unknown. Future studies investigating the factors associated with postsurgical readmission would benefit from the availability of data from remote automated monitoring systems to avoid these possible biases. Finally, while our results are commensurate with several previous studies, the area under the ROC curve of the prediction score was modest (C-statistic, 67.2%), highlighting that the predictive value of the model is limited in terms of distinguishing those patients who we will be readmitted versus those who will not and that readmission is a complex and multifactorial outcome. There may have been some unknown factors contributing to readmission that were not anticipated or captured during data collection.

## CONCLUSIONS

Readmission following surgery is a complex and challenging clinical problem. We identified risk factors associated with 30-day readmission following noncardiac surgery. Patients who experienced in-hospital stroke, episodes of postoperative tachycardia and bradycardia, decreased kidney function, preoperative tachycardia, and those treated for cancer in the previous 6 months had the highest risk of readmission. These factors, among the others identified, can help identify individuals who should be targeted for preventive measures, such as hospital-to-home remote automated monitoring and supportive care, to help reduce hospital readmission.

## ACKNOWLEDGMENT

All authors are The VISION Readmission Investigators.

## Supplementary Material


