# Supplementary material for: Risk Factors for Hospital Readmission Following Noncardiac Surgery: International Cohort Study
Source: Ann Surg Open. 2024 Apr 9;5(2):e417. doi: 10.1097/AS9.0000000000000417 (PMC11192008; doi:10.1097/AS9.0000000000000417)
Supplement: Supplementary file 1 [file as9-5-e417-s001.pdf]

Supplemental Digital Content (SDC)

Study Funding
Funding for this study came from numerous grants for VISION and its sub-studies including:
Canada: Canadian Institutes of Health Research - 7 grants; Heart and Stroke Foundation of Ontario – 2 grants; Academic Health Science Centres Alternative Funding Plan Innovation Fund Grant Ontario; Population Health Research Institute Grant; CLARITY Research Group Grant; McMaster University, Department of Anesthesiology – 2 grants; McMaster University, Department of Medicine – 2 grants; McMaster University, Department of Clinical Epidemiology and Biostatistics Grant; McMaster University, Division of Cardiology Grant; McMaster University, Department of Surgery, Surgical Associates Research Grant; Hamilton Health Science New Investigator Fund Grant; Hamilton Health Sciences Grant; Hamilton Health Sciences Summer Studentships – 6 grants; Ontario Ministry of Resource and Innovation Grant; Stryker Canada; Saint Joseph’s Healthcare, Department of Medicine – 2 grants; Father Sean O’Sullivan Research Centre – 2 grants; Roche Diagnostics Global Office – 5 grants; Canadian Network and Centre for Trials Internationally Grant; Winnipeg Health Sciences Foundation Operating Grant; University of Manitoba, Department of Surgery Research Grant – 2 grants; University of Manitoba, Faculty of Dentistry Operational Fund; University of Manitoba, Department of Anesthesia Grant; University of Manitoba, University Medical Group, Department of Surgery, start-up Fund; Diagnostic Services of Manitoba Research Grant; Manitoba Medical Services Foundation Grant; Manitoba Health Research Council Grant.
Australia: National Health and Medical Research Council Program Grant
Brazil: Projeto Hospitais de Excelência a Serviço do SUS (PROADI-SUS) grant from the Brazilian; Ministry of Health in Partnership with Hcor (Cardiac Hospital Sao Paulo-SP); National Council for Scientific and Technological Development (CNPq), grant from the
Brazilian Ministry of Science and Technology; National Institute for Health Technology Assessment - IATS/ CNPq grant; FIPE grant, Hospital de Clinicas de Porto Alegre
China: Public Policy Research Fund (CUHK-4002-PPR-3), Research Grant Council, Hong Kong, SAR; General Research Fund (461412), Research Grant Council, Hong Kong SAR; Australian and New Zealand College of Anaesthetists Grant (13/008), Melbourne, Australia
Colombia: School of Nursing, Universidad Industrial de Santander; Grupo de Cardiología Preventiva, Universidad Autónoma de Bucaramanga; Fundación Cardioinfantil – Instituto de Cardiología; Alianza Diagnóstica S.A.
France: Université Pierre et Marie Curie, Département d’anesthésie Réanimation, Pitié-Salpêtrière, Assistance Publique- Hôpitaux de Paris Grant
India: St. John's Medical College and Research Institute Grant, Division of Clinical Research and Training FGrant
Malaysia: University of Malaya Research Grant (RG302-14AFR); University of Malaya, Penyelidikan Jangka Pendek Grant (PJP)
Poland: Polish Ministry of Science and Higher Education (NN402083939) Grant.
South Africa: University of KwaZulu-Natal Grant
Spain: Instituto de Salud Carlos III (PI0790246); Fundació La Marató de TV3 (082330)
United States: American Heart Association Grant; Covidien Grant
United Kingdom: National Institute for Health Research (NIHR)

Role of the Funders/Sponsors: The VISION Study funding sources had no role in the design and conduct of the study; collection, management, analysis, and interpretation of the data; preparation or approval of the manuscript; or decision to submit the manuscript for publication.

SDC, Table 1: Participant demographic information by hospital readmission within 30 days of discharge following noncardiac surgery

	Patients readmitted (%)	Patients not readmitted (%)	p-value
Age group: 45-69 years	1725 (62.9)	23720 (69.9)	
70 years or older	1019 (37.1)	10193 (30.1)	<0.001
Median age (Q1-Q3)	64.7 (55.8-74.3)	63.0 (54.7-72.0)	<0.001
Female	1224 (44.6)	17152 (50.6)	
Male	1520 (55.4)	16761 (49.4)	<0.001
Region: North America	764 (27.8)	10287 (30.3)	
Europe	638 (23.2)	8275 (24.4)	
Australia	58 (2.1)	924 (2.7)	
South Africa	86 (3.1)	1016 (3.0)	
South America	307 (11.2)	5136 (15.1)	
Asia	891 (32.5)	8275 (24.4)	<0.001
Year of surgery			
2007	105 (3.8)	950 (2.8)	
2008	478 (17.4)	4789 (14.1)	
2009	388 (14.1)	5353 (15.8)	
2010	324 (11.8)	5077 (15.0)	
2011	398 (14.5)	4837 (14.3)	
2012	657 (23.9)	8564 (25.2)	
2013	394 (14.4)	4343 (12.8)	<0.001
Needs assistance with ADL	198 (7.2)	1444 (4.3)	
None needed	2546 (92.8)	32469 (95.7)	<0.001
Congestive heart failure	154 (5.6)	1075 (3.2)	
No history	2589 (94.4)	32817 (96.8)	<0.001
Cancer treatment in previous 6 months	618 (22.5)	4889 (14.4)	
No history	2126 (77.5)	29024 (85.6)	<0.001
Stroke	249 (96.1)	1996 (5.9)	
No history	2495 (90.9)	31917 (94.1)	<0.001
DVT/pulmonary embolism	124 (4.5)	1054 (3.1)	
No history	2617 (95.5)	32837 (96.9)	<0.001
Coronary artery disease	483 (17.6)	4185 (12.3)	
No history	2261 (82.4)	29728 (87.7)	<0.001
Atrial fibrillation	113 (4.1)	1021 (3.0)	
No history	2631 (95.9)	32892 (97.0)	<0.001
Peripheral vascular disease	289 (10.5)	2499 (7.4)	
No history	2455 (89.5)	31414 (92.6)	<0.001
Chronic obstructive pulmonary disease	274 (10.0)	2544 (7.5)	
No history	2470 (90.0)	31369 (92.5)	<0.001
Diabetes, history and/or insulin use	696 (25.4)	6827 (20.1)	
No history	2048 (74.6)	27086 (79.9)	<0.001
Tobacco use, ever	1423 (51.9)	15748 (46.4)	
No history	1321 (48.1)	18165 (53.6)	<0.001
Anemia[1]	1437 (52.4)	12957 (38.2)	
No anemia	1307 (47.6)	20956 (61.8)	<0.001
eGFR <30 mL/min/1·73[2] or on dialysis	155 (5.6)	901 (2.7)	
>30 / no dialysis	2589 (94.4)	33012 (97.3)	<0.001
Baseline hypertension (>160 mmHg)	516 (18.9)	5733 (17.0)	
≤160 mmHg	2216 (81.1)	28060 (83.0)	0.021
Baseline hypotension (<100 mmHg)	74 (2.7)	590 (1.7)	
≥100 mmHg	2670 (97.3)	33323 (98.3)	<0.001
Baseline bradycardia (<55/m)	72 (2.6)	1176 (3.5)	
≥55/m	2657 (97.4)	32529 (96.5)	0.030
Baseline tachycardia (>100/m)	213 (7.8)	1702 (5.0)	
≤100/m	2516 (92.2)	32003 (95.0)	<0.001
BMI: <18·5 (underweight)	159 (5.8)	1092 (3.2)	

	Patients readmitted (%)	Patients not readmitted (%)	p-value
≥18·5 (healthy, overweight, or obese)	2585 (94.2)	32821 (96.8)	<0.001
Surgery type: Orthopedic	338 (12.3)	5994 (17.7)	
Low risk	894 (32.6)	12377 (36.5)	
Neurological	163 (5.9)	1856 (5.5)	
Urological/gynecological	287 (10.5)	3544 (10.4)	
Multiple (>1 type)	95 (3.5)	985 (2.9)	
Vascular	210 (7.6)	2150 (6.3)	
Thoracic	94 (3.4)	932 (2.7)	
General	663 (24.2)	6075 (17.9)	<0.001
Hypoxemia (<90% for >20 m)	113 (4.1)	903 (2.7)	
≥90%	2631 (95.9)	33010 (97.3)	<0.001
Postoperative heart rate: Normal	947 (34.5)	14621 (43.1)	
Bradycardia (<55/m)	671 (24.4)	8820 (26.0)	
Tachycardia (>100/m)	855 (31.2)	8257 (24.3)	
Variable (episodes of both)	271 (9.9)	2215 (6.5)	<0.001
Postoperative systolic BP: Normal	340 (12.4)	5649 (16.7)	
Low (<100 mmHg)	1057 (38.5)	14271 (42.1)	
High (>160 mmHg)	358 (13.0)	4254 (12.5)	
Variable (episodes of both)	989 (36.0)	9739 (28.7)	<0.001
Stroke, in hospital	12 (0.4)	32 (0.1)	
None	2732 (99.6)	3381 (99.9)	<0.001
Pneumonia, in hospital	42 (1.5)	190 (0.6)	
None	2702 (98.5)	33723 (99.4)	<0.001
Sepsis, in hospital	108 (3.9)	713 (2.1)	
None	2636 (98.1)	33200 (97.9)	0.001
Major bleed, in hospital[2]	544 (19.8)	4149 (12.2)	
None	2200 (80.2)	29764 (87.8)	<0.001
DVT /pulmonary embolism, in hospital	17 (0.6)	138 (0.4)	
None	2727 (99.4)	33775 (99.6)	0.10
Length of stay ≤4 days after surgery	1144 (41.7)	19462 (57.4)	
>4 days	1600 (58.3)	14451 (42.6)	<0.001
Length of stay (median days-IQR)	6 (3-9)	4 (2-7)	<0.001
Discharged to nursing home	130 (4.7)	819 (2.4)	
Discharged home/other acute care	2614 (95.3)	33094 (97.6)	<0.001
MINS[3]	488 (17.8)	3786 (11.2)	
None	2256 (82.2)	30127 (88.8)	<0.001

ADL: activities of daily living (dressing-eating-ambulating-toileting-hygiene); BMI: body mass index (weight [kg] / height [m]^2); BP: blood pressure; DVT: deep vein thrombosis; eGFR: estimated glomerular filtration rate; IRR: incident rate ratio; m: minutes; mmHg: millimetres mercury;
* ≤0·05; ** ≤0·001

[1] Female: <120 g/L; Male: >140 g/L

[2] Bleeding that resulted in hemoglobin drop of ≥30 g/L and/or requires a transfusion or re-operation

[3] MINS: Myocardial injury after noncardiac surgery, within 30 days of surgery

SDC, Table 2: Risk factors associated with readmission within 30 days of discharge following non-cardiac surgery, population attributable fraction, and scores predictive of readmission

	Adjusted HR[1] (95% CI)	Population attributable fraction[1] %; (95% CI)	Points[2]
**Baseline demographics and medical history**			
Age ≥70 years	1.18 (1.09, 1.29)**	5.1 (2.7-7.4)	2
Cancer, treated in past 6 months	1.44 (1.30, 1.59)**	5.7 (4.1-7.3)	4
Tobacco use	1.22 (1.13, 1.33)**	8.0 (4.9-11.0)	2
DVT/pulmonary embolism	1.28 (1.07, 1.55)*	0.8 (0.1-1.5)	3
Congestive heart failure	1.23 (1.03, 1.46)*	0.9 (0.1-1.6)	2
Needs assistance with ADL	1.22 (1.04, 1.42)*	1.1 (0.2-1.9)	2
Peripheral vascular disease	1.18 (1.01, 1.39)*	1.5 (0.2-2.6)	2
Coronary artery disease	1.17 (1.05, 1.31)*	2.2 (0.7-3.6)	2
Stroke or transient ischemic attacks	1.15 (1.00, 1.32)*	1.1 (0.1-2.0)	2
Diabetes or blood glucose >10 mmol/L	1.10 (1.00, 1.20)*	1.4 (0.6-2.4)	1
Site: Canada/USA	Referent	0 (-0.1, 0)	
Europe	1.05 (0.94, 1.18)	0 (0, 0.1)	
Australia	0.84 (0.64, 1.10)	0 (0, 0.1)	
South Africa	1.12 (0.89, 1.41)	0.1 (0, 0.2)	
South America	0.99 (0.86, 1.14)	0 (-0.1, 0)	
Asia	1.47 (1.31, 1.64)**	7.6 (2.2, 12.9)	
**Baseline laboratory and physical measures**			
eGFR <30 mL/min/1.73^2 or on dialysis	1.47 (1.24, 1.75)**	1.5 (0.8-2.2)	5
Anemia[3]	1.28 (1.18, 1.39)**	9.7 (6.7-12.8)	3
Systolic BP 100-160 (normal)	Reference	--	--
<100 mmHg (low)	1.38 (1.09, 1.74)*	0.6 (0.2-1.1)	4
Heart rate: <55/minute (bradycardia)	Reference	--	0
55-100/minute	1.35 (1.06, 1.71)*	0.8 (0.2-1.3)	4
>100/minute (tachycardia)	1.57 (1.19, 2.08)*	0.9 (0-1.8)	6
BMI: ≥18.5	Reference	--	--
<18.5 (underweight)	1.11 (1.02, 1.21)*	0.9 (0.1-1.6)	1
**Type of surgery**			
Orthopedic	Reference	--	
General	1.86 (1.61, 2.16)**	9.4 (7.3-11.1)	
Neurological	1.78 (1.47, 2.16)**	2.2 (1.3-2.9)	
Multiple (>1 type at index surgery)	1.75 (1.39, 2.21)**	1.3 (0.7-1.8)	
Thoracic	1.68 (1.32, 2.15)**	1.2 (0.6-1.8)	
Urological/gynecological	1.70 (1.44, 2.01)**	3.5 (2.3-4.6)	
Low risk	1.61 (1.40, 1.85)**	10.1 (7.3-12.7)	
Vascular	1.43 (1.16, 1.77)**	2.0 (0.8-3.0)	
**Events during index hospitalization**			
Blood pressure (systolic): 100-160 mm Hg	Reference	--	--
<100 mm Hg	1.17 (1.03, 1.32)*	0.9 (0.2-1.9)	2
>160 mm Hg	1.16 (1.00, 1.36)	1.1 (0.5-1.8)	2
Variable (episodes of <100 & >160)	1.22 (1.07, 1.40)*	0.2 (0.1-0.8)	2
Heart rate: 55-100/min	Referent		
Tachycardia (>100/min)	1.22 (1.10-1.35)*	0.2 (0.1-0.8)	2
Bradycardia (<55/min)	1.15 (1.03-1.27)*	0.9 (0.2-1.9)	2
Variable (episodes of both)	1.42 (1.23-1.64)*	1.1 (0.7-1.6)	2
Stroke	2.21 (1.24, 3.94)*	0.2 (0-0.4)	12
Blood oxygen <90% for >20 minutes	1.36 (1.12, 1.65)*	0.9 (0.3-1.5)	4
Major bleeding[4]	1.19 (1.07, 1.33)*	2.6 (1.0-4.2)	2
**Other**			
Index length of stay: ≥4 days after surgery	1.38 (1.26, 1.50)**	13.3 (9.6-16.4)	4
Discharged to nursing home	1.61 (1.33, 1.95)**	1.5 (0.8-2.1)	6
MINS[5]	1.17 (1.05, 1.31)*	2.1 (0.7-3.5)	2

* ≤0.05; ** ≤0.001
ADL: activities of daily living; BP: blood pressure; DVT: deep vein thrombosis; eGFR: estimated glomerular filtration rate; IRR: incident rate ratio; mmHg: millimetres mercury
[1] Adjusted for region and variables in table
[2] Points predicted for each risk factor [1]
[3] Hemoglobin values of: Female: <120 g/L; Male: <140 g/L
[4] Bleeding that resulted in hemoglobin drop of ≥30 g/L and/or required a transfusion or re-operation
[5] MINS: Myocardial injury after noncardiac surgery, within 30 days of surgery

SDC, Table 3 Population attributable fraction associated with readmission within 30 days of discharge following non-cardiac surgery, by type of surgery

	Population attributable fractions (95% CI)[1]								
	**All participants (N=36657)**	**Orthopedic (N=6332)**	**Low risk (N=13271)**	**Neurological (N=2019)**	**Urology/ gynecology (N=3804)**	**Multiple (N=1080)**	**Vascular (N=2360)**	**Thoracic (N=1026)**	**General (N=6738)**
**Baseline demographics and medical history**									
Age ≥70 years	5.1 (2.7, 7.4)*	9.5 (-4.1, 21.3)	7.7 (3.2, 12.0)*	-2.2 (-12.3, 7.0)	2.3 (-6.6, 10.5)	-2.0 (-17.6, 11.6)	7.1 (-7.0, 19.4)	7.3 (-8.0, 20.5)	2.6 (-3.3, 8.2)
Cancer, treated in past six months	5.7 (4.1, 7.3)*	1.6 (-0.5, 3.7)	8.8 (5.7, 11.8)*	4.7 (-1.6, 10.7)	9.5 (1.4, 17.0)*	0.9 (-14.3, 14.1)	2.6 (-0.6, 5.7)	0.5 (-21.3, 18.3)	4.2 (-1.3, 9.4)
Tobacco use	8.0 (4.9, 11.0)*	4.3 (-5.6, 13.3)	11.1 (5.3, 16.5)*	-3.3 (-21.1, 18.9)	18.2 (6.7, 28.2)*	5.7 (-14.6, 22.5)	11.2 (-28.0, 38.4)	-4.3 (-39.0, 21.6)	7.8 (-0.4, 15.3)
DVT/pulmonary embolism	0.8 (0.1, 1.5)*	0.1 (-2.4, 2.6)	1.2 (-0.1, 2.5)	2.4 (-1.3, 6.1)	1.2 (-1.2, 3.6)	1.3 (-3.7, 6.0)	0.8 (-2.7, 4.1)	-1.2 (-3.5, 1.1)	1.2 (-0.6, 3.0)
Congestive heart failure	0.9 (0.1, 1.6)*	2.1 (-1.5, 5.6)	1.7 (0.1, 3.4)*	0.4 (-2.6, 3.2)	0 (-2.6, 2.5)	2.4 (-2.6, 7.2)	0.8 (-4.2, 5.6)	1.9 (-2.5, 6.1)	-0.4 (-2.0, 1.1)
Needs assistance with ADL	1.1 (0.2, 1.9)*	3.9 (-1.5, 8.9)	0.4 (-1.6, 2.3)	0.9 (-4.4, 6.0)	-0.6 (-2.6, 1.5)	-1.9 (-4.9, 0.9)	1.1 (-2.7, 4.8)	-0.9 (-4.2, 2.4)	1.6 (-0.1, 3.4)
Peripheral vascular disease	1.5 (0.2, 2.6)*	4.6 (0.6, 8.5)*	0.4 (-1.4, 2.1)	1.4 (-1.4, 4.1)	1.0 (-1.2, 3.2)	-0.3 (-5.1, 4.3)	14.9 (-7.5, 32.7)	0.2 (-3.8, 4.0)	0.1 (-1.4, 1.5)
Coronary artery disease	2.2 (0.7, 3.6)*	1.4 (-4.3, 6.9)	3.0 (0.1, 5.8)*	2.2 (-4.6, 8.5)	4.5 (-1.0, 9.7)	-0.6 (-11.2, 9.0)	2.8 (-10.3, 14.3)	-3.3 (-12.2, 4.9)	2.9 (-0.3, 6.0)
Stroke or transient ischemic attacks	1.1 (0.1, 2.0)*	3.4 (-1.2, 7.8)	3.0 (0.9, 5.0)*	0.6 (-5.0, 6.0)	2.3 (-1.2, 5.8)	1.4 (-4.1, 6.6)	-4.5 (-11.4, 2.1)	-1.6 (-6.3, 2.9)	-0.4 (-2.4, 1.5)
**Baseline laboratory and physical measures**									
eGFR <30 mL/min/1.73[2] or on dialysis	1.5 (0.8, 2.2)*	0.5 (-1.9, 2.8)	2.0 (0.2, 3.9)*	0.5 (-1.2, 2.2)	3.0 (-0.6, 6.5)	3.2 (-1.7, 7.8)	4.8 (0.7, 8.8)*	-1.1 (-1.8, -0.5)	1.1 (-0.5, 2.7)
Anemia[3]	9.7 (6.7, 12.8)*	6.3 (-6.2, 17.4)	16.2 (9.3, 22.6)*	4.8 (-6.3, 14.8)	7.3 (-5.3, 18.5)	-2.5 (-26.6, 17.0)	14.0 (-0.8, 26.6)	2.4 (-18.2, 19.4)	10.4 (2.1, 18.1)*
Systolic BP 100-160	Referent	Referent	Referent	Referent	Referent	Referent	Referent	Referent	Referent
<100 mmHg	0.6 (0.2, 1.1)*	-0.5 (-1.1, 1.0)	0.4 (-0.6, 1.4)	0 (-2.1, 2.1)	1.3 (-0.6, 3.3)	0.5 (-2.5, 3.5)	1.5 (-1.1, 4.0)	3.9 (-1.4, 8.9)	0.6 (-0.9, 2.1)
Heart rate: <55/minute	-0.9 (-1.5, -0.3)*	-2.4 (-3.4, -1.4)*	-0.9 (-2.0, -0.1)*	-0.6 (-3.7, 2.3)	-0.5 (-2.7, 1.6)	-1.8 (-4.1, 0.5)	-0.4 (-3.4, 2.6)	-0.1 (-3.0, 2.7)	-0.5 (-1.8, 0.8)
55-100/minute	Referent	Referent	Referent	Referent	Referent	Referent	Referent	Referent	Referent
>100/minute	0.9 (0, 1.8)*	0.4 (-2.6, 3.3)	1.9 (-0.1, 3.8)	-0.6 (-3.9, 2.5)	1.9 (-1.1, 4.8)	-3.3 (-7.7, 1.0)	2.7 (-0.6, 5.9)	-2.3 (-7.5, 2.6)	1.5 (-0.7, 3.7)
BMI: ≥18.5	Referent	Referent	Referent	Referent	Referent	Referent	Referent	Referent	Referent
<18.5 (underweight)	0.9 (0.1, 1.6)*	3.1 (-0.1, 6.2)	0.8 (-0.8, 2.5)	-1.3 (-3.2, 5.1)	-0.1 (-2.2, 1.8)	3.8 (-2.0, 9.3)	0.5 (-2.3, 3.2)	7.5 (-1.4, 15.6)	0.4 (-1.5, 2.4)
**Events during index hospitalization**									
Heart rate: 55-100/min	Referent								
<55/min	3.0 (1.0, 4.8)*	3.2 (-3.0, 9.1)	1.4 (-2.4, 5.2)	14.3 (1.9, 25.2)*	9.1 (0.9, 16.5)*	5.0 (-9.4, 17.4)	1.8 (-9.9, 12.2)	3.7 (-9.2, 15.0)	3.1 (-1.7, 7.6)
>100/min	5.0 (2.7, 7.1)*	7.4 (-1.1, 15.1)	5.6 (1.0, 10.0)*	12.4 (0.8, 22.7)*	6.4 (-1.4, 13.6)	5.2 (-16.1, 22.5)	-0.4 (-9.0, 7.4)	10.9 (-10.2, 28.1)	2.7 (-5.1, 9.8)
Variable (both)	2.6 (1.5, 3.5)*	3.1 (-0.1, 6.3)	1.7 (0, 3.4)*	6.3 (0.1, 12.0)*	4.6 (0.5, 8.4)*	5.3 (-5.4, 15.0)	5.1 (-1.1, 10.9)	3.2 (-5.2, 10.9)	2.1 (-1.1, 5.1)
Stroke	0.2 (0, 0.4)*	0.7 (-0.3, 1.7)	0 (-0.2, 0.2)	3.4 (0.1, 6.5)*	-0.1 (-0.2, -0.1)	---	0.2 (-0.8, 1.1)	0 (-0.1, 0)	-0.1 (-0.1, 0)
Blood oxygen <90% for >20 minutes	0.9 (0.3, 1.5)*	2.3 (-0.4, 4.9)	1.1 (0.0, 2.1)*	1.1 (-1.7, 3.9)	0.3 (-2.0, 2.5)	2.8 (-4.2, 9.4)	1.2 (-1.9, 4.3)	0.1 (-5.1, 5.0)	0.5 (-1.3, 2.2)
Major bleeding[3]	2.6 (1.0, 4.2)*	9.8 (1.0, 17.8)*	2.3 (0, 4.4)*	-2.1 (-9.4, 4.5)	4.3 (-1.9, 10.1)	18.3 (1.9, 32.0)*	0.5 (-9.1, 9.2)	0 (-9.5, 8.7)	3.3 (-0.9, 7.3)

Population attributable fractions (95% CI)[1]									
	**All participants (N=36657)**	**Orthopedic (N=6332)**	**Low risk (N=13271)**	**Neurological (N=2019)**	**Urology/ gynecology (N=3804)**	**Multiple (N=1080)**	**Vascular (N=2360)**	**Thoracic (N=1026)**	**General (N=6738)**
**Other**									
Length of stay ≥4 days after surgery	13.3 (9.6, 16.4)*	6.1 (-12.8, 21.9)	12.4 (7.0, 17.5)*	19.0 (-0.1, 34.5)	10.8 (-0.5, 20.9)	33.5 (5.8, 53.0)*	1.0 (-20.9, 19.0)	1.8 (-23.3, 21.9)	29.4 (18.4, 39.0)*
Discharged to nursing home	1.5 (0.8, 2.1)*	0.5 (-3.5, 4.3)	2.2 (0.8, 3.5)*	-0.1 (-3.3, 3.0)	3.6 (0.9, 6.2)*	1.3 (-2.5, 4.9)	-0.5 (-2.8, 1.7)	3.5 (-0.7, 7.5)	1.0 (-0.4, 2.4)
MINS[4]	2.1 (0.7, 3.5)*	6.7 (0.2, 12.8)*	1.7 (-1.2, 4.4)	-0.1 (-6.4, 5.8)	1.1 (-4.5, 6.4)	6.7 (-4.2, 16.5)	8.0 (-1.9, 17.0)	0.6 (-10.5, 10.6)	1.3 (-2.1, 4.6)

* ≤0.05

ADL: activities of daily living; BP: blood pressure; DVT: deep vein thrombosis; eGFR: estimated glomerular filtration rate; IRR: incident rate ratio; min: minute; mmHg: millimetres mercury

[1] Adjusted for region and variables in table

[2] Hemoglobin values of: Female: <120 g/L; Male: <140 g/L

[3] Bleeding that resulted in hemoglobin drop of ≥30 g/L and/or required a transfusion or re-operation

[4] MINS: Myocardial injury after noncardiac surgery, within 30 days of surgery

SDC, Table 4: Population attributable fraction associated with readmission within 30 days of discharge following non-cardiac surgery, by study region

	Population attributable fractions (95% CI)[1], by study region						
	**All participants (N=36657)**	**North America (N=11051)**	**Europe (N=8913)**	**Australia (N=982)**	**Africa (N=1102)**	**South America (N=5443)**	**Asia (N=9166)**
**Baseline demographics and medical history**							
Age ≥70 years	5.1 (2.7, 7.4)*	0.4 (-5.3, 5.7)	3.3 (-3.2, 9.4)	20.2 (-1.9, 37.5)	-8.8 (-18.8, 0.4)	8.7 (0.1, 16.7)*	9.6 (4.9, 14.0)*
Cancer, treated in past six months	5.7 (4.1, 7.3)*	7.9 (3.7, 11.9)*	7.5 (3.2, 11.7)*	-1.2 (-8.5, 5.7)	-7.5 (-14.0, -1.3)*	9.9 (3.6, 15.8)*	6.0 (3.2, 8.7)*
Tobacco use	8.0 (4.9, 11.0)*	9.3 (0.4, 17.3)*	11.3 (1.1, 20.5)*	11.8 (-19.4, 34.8)	20.9 (0.2, 37.3)*	10.6 (-0.2, 20.3)	3.9 (-0.3, 7.9)
DVT/pulmonary embolism	0.8 (0.1, 1.5)*	1.5 (-0.5, 3.4)	1.8 (-0.0, 3.6)	5.1 (-2.6, 12.3)	2.9 (-2.4, 7.9)	0.1 (-1.6, 1.8)	-0.2 (-0.8, 0.4)
Congestive heart failure	0.9 (0.1, 1.6)*	0.7 (-0.1, 2.4)	1.5 (-0.7, 3.7)	4.4 (-4.3, 12.5)	0.1 (-2.8, 3.0)	0.3 (-2.6, 3.1)	1.1 (-0.2, 2.4)
Needs assistance with ADL	1.1 (0.2, 1.9)*	0.0 (-1.6, 1.6)	1.6 (-0.5, 3.6)	5.3 (-1.9, 12.0)	5.3 (-4.6, 14.3)	2.2 (-1.2, 5.4)	0.8 (-1.0, 2.6)
Peripheral vascular disease	1.5 (0.2, 2.6)*	2.4 (0.2, 4.5)*	-2.7 (-8.4, 2.6)	5.9 (-5.5, 16.0)	6.7 (-11.0, 21.5)	2.1 (-1.3, 5.4)	1.1 (-0.3, 2.6)
Coronary artery disease	2.2 (0.7, 3.6)*	1.4 (-2.3, 5.0)	6.3 (2.2, 10.2)*	0.7 (-18.3, 16.7)	8.1 (-1.1, 16.5)	2.8 (-2.0, 7.4)	1.1 (-1.3, 3.6)
Stroke or transient ischemic attacks	1.1 (0.1, 2.0)*	2.2 (-0.4, 2.1)	1.3 (-1.2, 3.8)	5.9 (-5.7, 16.2)	-5.1 (-8.4, -2.0)*	-0.9 (-3.5, 1.6)	1.0 (-1.2, 3.1)
**Baseline laboratory and physical measures**							
eGFR <30 mL/min/1.73[2] or on dialysis	1.5 (0.8, 2.2)*	0.8 (-0.4, 2.1)	3.2 (1.3, 5.0)*	6.5 (-1.7, 14.0)	3.8 (-2.0, 9.3)	1.0 (-1.9, 3.7)	1.4 (-0.2, 2.9)
Anemia[3]	9.7 (6.7, 12.8)*	9.1 (2.9, 14.9)*	9.5 (2.4, 16.2)*	-24.0 (-53, -0.4)*	11.6 (-17.7, 33.6)	10.7 (-0.4, 20.3)	15.2 (7.4, 22.4)*
Systolic BP 100-160	Referent						
<100 mmHg	0.6 (0.2, 1.1)*	1.9 (0.6, 3.2)*	0.3 (-0.1, 1.6)	-3.6 (-5.8, -1.5)*	0.3 (-2.7, 3.1)	0.8 (-1.2, 2.9)	0.2 (-0.5, 0.9)
Heart rate: <55/minute	-0.9 (-1.5, -0.3)*	-0.2 (-1.5, 1.1)	-1.6 (-3.0, -0.2)*	-1.9 (-3.0, -0.7)*	-2.0 (-5.5, 1.4)	-1.6 (-3.3, 0.1)	-0.6 (-1.5, 0.3)
55-100/minute	Referent	Referent	Referent	Referent	Referent	Referent	Referent
>100/minute	0.9 (0, 1.8)*	0.2 (-1.5, 1.9)	1.3 (-0.3, 2.8)	3.5 (-4.0, 10.4)	7.4 (0.3, 14.0)*	0.1 (-2.5, 2.8)	0.7 (-1.6, 3.1)
BMI: ≥18.5	Referent						
<18.5 (underweight)	0.9 (0.1, 1.6)*	-0.1 (-1.0, 0.8)	1.5 (0.2, 2.7)*	-2.6 (-3.8, -1.5)*	0.8 (-3.8, 5.3)	3.0 (0.3, 5.7)*	0.6 (-1.7, 2.8)
**Type of surgery**							
Orthopedic	Referent						
General	9.4 (7.3, 11.1)*	17.7 (13.5, 21.7)*	9.5 (4.4, 14.3)*	1.4 (-25.4, 22.5)	4.7 (-1.0, 10.0)	-7.8 (-21.0, 3.9)	9.1 (5.2, 12.8)*
Neurological	2.2 (1.3, 2.9)*	4.9 (2.8, 7.0)*	2.6 (0.8, 4.4)*	-8.0 (-15.6, -0.9)*	4.5 (-1.5, 10.2)	-0.4 (-3.2, 2.3)	1.8 (0.4, 3.2)*
Multiple (>1 type)	1.3 (0.7, 1.8)*	3.8 (2.0, 5.5)*	0.2 (-0.6, 1.1)	-1.3 (-5.5, 2.7)	-0.4 (-0.7, -0.2)*	-0.3 (-1.6, 1.0)	1.6 (0.3, 2.9)*
Thoracic	1.2 (0.6, 1.8)*	2.9 (1.1, 4.7)*	-0.2 (-0.7, 0.4)	0.7 (-2.2, 3.5)	3.7 (-0.5, 7.7)	-2.9 (-6.5, 0.6)	1.7 (0.6, 2.8)*
Urology/gynecology	3.5 (2.3, 4.6)*	6.1 (3.5, 8.7)*	3.8 (1.3, 6.2)*	-7.0 (-31.8, 13.1)	-1.4 (-6.4, 3.3)	-6.7 (-13.1, -0.7)*	5.2 (2.9, 7.5)*
Low risk	10.1 (7.3, 12.7)*	8.3 (5.0, 11.6)*	10.5 (3.8, 16.7)*	-8.5 (-37.2, 14.1)	13.6 (-13.4, 34.2)	-12.1 (-25.0, -0.5)*	22.2 (14.1, 29.7)*
Vascular	2.0 (0.8, 3.0)*	3.2 (1.3, 5.0)*	5.6 (0.6, 10.4)*	-3.2 (-15.2. 7.6)	7.7 (-2.5, 16.9)	-1.3 (-4.7, 1.9)	-0.5 (-0.8, 0.7)
**Events during index hospitalization**							
Heart rate: 55-100/min	Referent						
<55/min	3.0 (1.0, 4.8)*	5.0 (-0.1, 9.7)	-0.5 (-6.0, 4.8)	-0.4 (-15.0, 12.3)	0.5 (-13.0, 12.4)	3.1 (-2.6, 8.6)	4.4 (1.1, 7.6)*

>100/min	5.0 (2.7, 7.1)*	5.0 (-1.0, 10.7)	1.9 (-2.3, 5.9)	9.4 (-10.4, 25.7)	-38.8 (-69.8, -13.4)*	8.3 (2.1, 14.2)*	11.4 (6.2, 16.4)*
Variable (both)	2.6 (1.5, 3.5)*	2.5 (-0.1, 5.1)	0.4 (-1.5, 2.3)	-3.7 (-10.2, 2.4)	3.8 (-5.7, 12.5)	2.4 (-0.2, 5.0)	5.6 (3.2, 7.8)*
Stroke	0.2 (0, 0.4)*	0.0 (-1.1, 2.3)	-0.1 (-0.1, -0.1)*	Not calculable	-0.4 (-0.8, 0)	0.5 (-0.1, 1.1)	0.7 (0.1, 1.2)*
Blood oxygen <90% for >20 minutes	0.9 (0.3, 1.5)*	0.6 (-1.1, 2.3)	0.4 (-0.6, 1.5)	-1.1 (-1.8, -0.3)*	-2.2 (-3.4, -0.1)*	7.8 (2.8, 11.4)*	0.2 (-0.2, 0.7)
Major bleeding[3]	2.6 (1.0, 4.2)*	3.3 (-0.8, 7.2)	3.6 (-0.3, 7.4)	3.3 (-6.5, 12.1)	7.1 (-5.0, 17.8)	2.9 (-1.0, 6.6)	3.3 (0.1, 6.5)*
**Other**							
Length of stay ≥4 days after surgery	13.3 (9.6, 16.4)*	16.0 (8.5, 22.8)*	17.6 (7.8, 26.4)*	15.6 (-16.8, 39.1)	17.3 (-7.7, 36.5)	6.9 (-3.8, 16.5)	18.9 (11.2, 25.8)*
Discharged to nursing home	1.5 (0.8, 2.1)*	0.3 (-0.9, 1.5)	0.7 (-1.0, 2.3)*	-1.6 (-9.0, 5.2)	4.1 (-0.9, 8.8)	2.0 (-0.1, 4.0)	3.4 (1.9, 4.9)*
MINS[4]	2.1 (0.7, 3.5)*	3.7 (0.1, 7.1)*	3.1 (-0.4, 6.6)	-1.7 (-16.0, 10.8)	-13.6 (-26.3, -2.2)*	7.5 (1.6, 13.0)*	1.3 (-1.5, 3.9)

* ≤0.05

ADL: activities of daily living; BP: blood pressure; DVT: deep vein thrombosis; eGFR: estimated glomerular filtration rate; IRR: incident rate ratio; min: minute; mmHg: millimetres mercury

[1] Adjusted for variables in table

[2] Hemoglobin values of: Female: <120 g/L; Male: <140 g/L

[3] Bleeding that resulted in hemoglobin drop of ≥30 g/L and/or required a transfusion or re-operation

[4] MINS: Myocardial injury after noncardiac surgery, within 30 days of surgery

SDC, Table 5: Population attributable fraction associated with readmission within 30 days of discharge following non-cardiac surgery, by year of surgery

Population attributable fractions (95% CI)[1], by year of surgery				
	**All participants (N=36657)**	**2007-2009 (N=12063)**	**2010-2011 (N=10636)**	**2012-2013 (N=13958)**
**Baseline demographics and medical history**				
Age ≥70 years	5.1 (2.7, 7.4)*	9.9 (4.5, 15.0)*	5.2 (0, 10.2)*	1.6 (-2.5, 5.6)
Cancer, treated in past six months	5.7 (4.1, 7.3)*	3.6 (1.3, 5.9)*	14.0 (9.7, 18.0)*	5.1 (1.5, 8.5)*
Tobacco use	8.0 (4.9, 11.0)*	6.5 (0.7, 12.1)*	11.7 (4.3, 18.4)*	7.7 (1.2, 13.9)*
DVT/pulmonary embolism	0.8 (0.1, 1.5)*	1.0 (0.2, 2.2)*	1.4 (-0.1, 2.8)	0.3 (-1.0, 1.7)
Congestive heart failure	0.9 (0.1, 1.6)*	2.4 (0.6, 4.1)*	0.4 (-1.3, 2.1)	0.1 (-1.2, 1.2)
Needs assistance with ADL	1.1 (0.2, 1.9)*	1.6 (-0.3, 3.4)	0.8 (-1.3, 2.9)	1.5 (0.2, 2.9)*
Peripheral vascular disease	1.5 (0.2, 2.6)*	0.8 (-1.1, 2.7)	0.9 (-2.5, 4.2)	2.5 (-0.1, 5.1)
Coronary artery disease	2.2 (0.7, 3.6)*	1.1 (-.5, 3.7)	6.0 (2.3, 9.6)*	2.5 (-0.4, 5.2)
Stroke or transient ischemic attacks	1.1 (0.1, 2.0)*	1.4 (-0.8, 3.5)	1.7 (-0.5, 3.9)	0.6 (-1.0, 2.3)
**Baseline laboratory and physical measures**				
eGFR <30 mL/min/1.73[2] or on dialysis	1.5 (0.8, 2.2)*	0.7 (-0.7, 2.1)	2.1 (0.4, 3.8)*	2.3 (0.9, 3.7)*
Anemia[3]	9.7 (6.7, 12.8)*	9.4 (3.0, 15.4)*	14.4 (7.1, 21.1)*	11.2 (5.3, 16.7)*
Systolic BP 100-160	Referent	Referent	Referent	Referent
<100 mmHg	0.6 (0.2, 1.1)*	1.0 (-0.1, 2.0)	0.3 (-0.7, 1.4)	0.8 (-0.1, 1.6)
Heart rate: <55/minute	-0.9 (-1.5, -0.3)*	-0.4 (-1.6, 0.7)	-1.4 (-2.3, -0.4)*	-0.9 (-2.0, 0.1)
55-100/minute	Referent	Referent	Referent	Referent
>100/minute	0.9 (0, 1.8)*	0.9 (-0.9, 2.8)	2.2 (0.1, 4.3)*	0.2 (-1.3, 1.8)
BMI: ≥18.5	Referent	Referent	Referent	Referent
<18.5 (underweight)	0.9 (0.1, 1.6)*	1.5 (-0.3, 3.3)	0.1 (-1.3, 1.4)	0.9 (-0.3, 2.2)
**Type of surgery**				
Orthopedic	Referent	Referent	Referent	Referent
General	9.4 (7.3, 11.1)*	9.3 (5.4, 13.1)*	6.1 (1.6, 10.4)*	15.7 (11.8, 19.4)*
Neurological	2.2 (1.3, 2.9)*	2.6 (1.1, 4.2)*	2.5 (0.6, 4.4)*	2.4 (0.8, 3.8)*
Multiple (>1 type)	1.3 (0.7, 1.8)*	1.7 (0.3, 3.1)*	1.4 (0.2, 2.6)*	1.1 (0.1, 2.0)*
Thoracic	1.2 (0.6, 1.8)*	0.8 (-0.1, 1.8)	0.5 (-1.1, 2.0)	2.3 (1.0, 3.6)*
Urology/gynecology	3.5 (2.3, 4.6)*	2.4 (0.4, 4.3)*	3.0 (0.1, 5.9)*	6.5 (4.2, 8.8)*
Low risk	10.1 (7.3, 12.7)*	11.9 (6.2, 17.2)*	8.0 (0.4, 14.9)*	14.1 (9.6, 18.4)*
Vascular	2.0 (0.8, 3.0)*	1.0 (-0.3, 2.3)	2.4 (-0.3, 5.1)	3.8 (1.2, 6.3)*
**Events during index hospitalization**				
Heart rate: 55-100/min	Referent	Referent	Referent	Referent
<55/min	3.0 (1.0, 4.8)*	-0.3 (-0.4, 3.6)	4.0 (-0.1, 7.9)	4.5 (0.7, 8.2)*
>100/min	5.0 (2.7, 7.1)*	4.7 (-0.6, 9.6)	3.9 (-1.7, 9.1)	5.9 (1.8, 9.9)*
Variable (both)	2.6 (1.5, 3.5)*	3.4 (1.0, 5.7)*	1.3 (-0.6, 3.2)	2.4 (0.4, 4.3)*
Stroke	0.2 (0, 0.4)*	0.6 (0.1, 1.1)*	0.1 (-0.2, 4.7)	-0.1 (-0.1, -0.1)*
Blood oxygen <90% for >20 minutes	0.9 (0.3, 1.5)*	0.5 (-0.5, 1.6)	0.8 (-0.5, 2.1)	1.9 (0.5, 3.3)*
Major bleeding[3]	2.6 (1.0, 4.2)*	1.9 (-1.6, 5.2)	1.5 (-2.3, 5.1)	5.0 (2.1, 7.8)*
**Other**				
Length of stay ≥4 days after surgery	13.3 (9.6, 16.4)*	22.3 (15.1, 28.9)	10.6 (2.3, 18.1)*	13.5 (6.9, 19.6)*
Discharged to nursing home	1.5 (0.8, 2.1)*	-0.3 (-1.6, 1.0)	1.0 (-0.2, 2.2)	3.5 (2.1, 4.9)*
MINS[4]	2.1 (0.7, 3.5)*	3.2 (0.9, 5.4)*	1.2 (-2.4, 4.7)	3.4 (0.2, 6.6)*

* ≤0.05

ADL: activities of daily living; BP: blood pressure; DVT: deep vein thrombosis; eGFR: estimated glomerular filtration rate; IRR: incident rate ratio; min: minute; mmHg: millimetres mercury

[1] Adjusted for region and variables in table

[2] Hemoglobin values of: Female: <120 g/L; Male: <140 g/L

[3] Bleeding that resulted in hemoglobin drop of ≥30 g/L and/or required a transfusion or re-operation

[4] MINS: Myocardial injury after noncardiac surgery, within 30 days of surgery

SDC, Table 6: Model-based risk of hospital readmission within 30 days of discharge following non-cardiac surgery

Point total*	Model-based risk [1] (%)
7	0.3
8	0.9
9	1.5
10	2.1
11	2.7
12	3.3
13	3.9
14	4.5
15	5.1
16	5.7
17	6.3
18	6.9
19	7.5
20	8.1
21	8.7
22	9.3
23	9.9
24	10.5
25	11.1
26	11.7
27	12.3
28	12.9
29	13.5
30	14.1

*Points start at 7 as fewer points did not increase the risk of readmission based on the model
